# Robotic-Assisted Urologic Surgery in Infants: Positioning, Trocar Placement, and Physiological Considerations

**DOI:** 10.3389/fped.2018.00411

**Published:** 2019-01-07

**Authors:** Soo Jeong Kim, John S. Barlog, Ardavan Akhavan

**Affiliations:** ^1^Institute for Pediatric Urology, Komansky Children's Hospital, New York Presbyterian-Weill Cornell, New York, NY, United States; ^2^SUNY Downstate Medical Center, Brooklyn, NY, United States

**Keywords:** robotic-assisted laparoscopic surgery, surgery in infants, positioning and orientation, trocar placement, robotic-assisted laparoscopic pyeloplasty, robotic-assisted laparoscopic reimplant, robotic-assisted laparoscopic nephrectomy

## Abstract

Pediatric robotic-assisted laparoscopic procedures are becoming increasingly common. They have been shown to be safe in younger patients, including infants. Successful adoption of robotic-assisted surgery in infants requires an understanding of the technical factors unique to this patient population. This review will delineate the specific considerations to safely perform robotic-assisted laparoscopic procedures in infants, including physiological changes associated with pneumoperitoneum in infants, positioning, trocar placement, and docking.

## Introduction

Since the introduction of laparoscopy in children (1), indications have expanded from simple diagnostic procedures to complex, reconstructive surgeries. While the most commonly performed pediatric robotic-assisted laparoscopic procedures are pyeloplasty, ureteral reimplantation, nephrectomy, and heminephrectomy (2), more complex procedures like appendicovesicostomy, bladder neck reconstruction, and augmentation cystoplasty are being performed by early adopters. Robotic surgical assistance has potentiated this adoption by adding high definition three-dimensional (stereoscopic) visualization as well as superior articulation, thereby allowing for more accurate movements and improved ergonomics (3). In addition, the magnified image can be combined with tremor filtration and motion scaling, allowing for delicate motions in small areas, which is particularly beneficial in pediatric cases. These benefits have propelled the adoption of robotically-assisted cases by 17.4% per year (*P* < 0.0001) between 2008 and 2013 (2). Furthermore, as experience has grown, the lower age of patients suitable for robotic surgery has declined, and several studies have demonstrated the safety of using robotic-assisted surgery in infants (4, 5).

Successful adoption of robotic-assisted surgery in infants requires understanding of the technical factors unique to this patient population. This review focuses on both the physiological changes associated with pneumoperitoneum in infants, as well as the positioning, trocar placement, and docking considerations that must be addressed in order to successfully execute robotic surgery in the youngest patients.

## Physiological Considerations

Robotic-assisted laparoscopic surgery requires distension of the peritoneal cavity, and given the infant anatomy, there are several physiological factors to consider during robotic-assisted laparoscopic surgery in infants.

### Respiratory

Increased intra-abdominal pressurefrom pneumoperitoneum can exert pressure on the lungs by cephalad displacement of the diaphragm, which decreases total lung capacity, and increases the peak inspiratory pressure, further decreasing the functional residual capacity. The Trendelenberg position can exacerbate these changes. Using CO_2_ to maintain pneumoperitoneum also leads to direct increases in CO_2_ absorption. These changes lead to acidosis, which is pressure-dependent. An increase in ventilatory minute volume is necessary to limit the respiratory perturbations following insufflation (6). This increase in CO_2_ has been shown to be inversely correlated with age, possibly because the peritoneum of infants is relatively larger, and better perfused (7). Also, unlike adults, where the level of CO_2_ tends to plateau during surgery, in infants the level of CO_2_ continues to rise with the duration of surgery (6).

### Cardiovascular

Pneumoperitoneum also exerts cardiovascular effects due to an increase in intraabdominal pressure, peritoneal absorption of CO_2_, and stimulation of the neurohumoral vasoactive system. In young children aged 6–36 months, an intraabdominal pressure of 10 mmHg or greater (i.e., higher than the right atrial pressure) causes a decrease in venous return, right ventricular cardiac output, and left ventricular preload and cardiac output (8). In addition, increased intraabdominal pressure may cause a catecholamine release which could contribute to increased mean arterial pressure and systemic vascular resistance (9). Pediatric patients can also develop bradycardia due to a robust vagal reflex induced by pneumoperitoneum, which may require emergent desufflation (10, 11). In a prospective study evaluating 33 pediatric patients aged 1–14 years (median 5 years) undergoing laparoscopic surgery, no significant cardiovascular changes occurred during CO_2_ insufflation if the intraabdominal pressure was maintained lower than 10 mmHg (12).

Positioning for robotic surgery can also affect cardiovascular status. The Trendelenberg position, for instance, can increase venous return to the heart and therefore cardiac filling pressure, while reverse-Trendelenberg reduces the two (13).

### Renal

In adults, pneumoperitoneum is associated with a pressure-dependent, reversible decrease in renal blood flow, glomerular filtration, and resultant urinary output (14). When 8 patients aged 0–12 months with normal baseline renal function underwent laparoscopy with intraabdominal pressure maintained at 8 mmHg, 88% developed anuria (15). The anuria was reversible, however, with urine output significantly increased postoperatively, and maximum values were evident at 5 h after desufflation (15).

Pneumoperitoneum causes renal changes via stimulation of the renin-angiotensin-aldosterone system (increase in renin and subsequent aldosterone secretion) and excretion of anti-diuretic hormone. These hormonal changes contribute to salt and water retention with oliguria (16).

### Insufflation

In addition to the physiologic changes as outlined above, mean postoperative pain score and requirement for analgesia have been shown to be positively correlated with pneumoperitoneal pressure during laparoscopic renal surgery in infants (17). Therefore, we recommend that the lowest possible insufflation pressure be used in infants [8–12 mmHg]. For infants and young children, we recommend beginning the insufflation with the lowest possible flow rate, in order to allow for any adverse physiological changes to be identified and immediately reversed, if necessary. Once the target pressure has been reached, the flow may be increased for the remainder of the case.

### Other Anesthetic Concerns

There are several additional anesthesia-related considerations. First, no nitric oxide should be used in infants during robotic surgery as nitric oxide can lead to bowel distension, obscuring the surgical field. Second, intubation with an endotracheal tube is recommended as laryngeal mask airways will leak at a pressure less than required for robotic surgery. Third, patients should be paralyzed to allow for increased intraabdominal distension. Fourth, the robot is cumbersome and positioning for robotic surgery frequently requires the head of the patient to be far from the anesthesiologist. Anesthesiologists should know how to access the patient in case of emergency, and a plan should be in place to quickly undock if necessary. Lastly, aerosolized bupivacaine can be used to decrease the length of hospitalization, postoperative, and total postoperative narcotic requirements (18).

### Physiologic Conclusions

Given the increased time-dependent physiological effects of pneumoperitoneum in infants, providers should take an honest assessment of their operative times and limit patient selection to older children until they are experienced enough to reliably execute procedures within a reasonable length of console time. Furthermore, surgeons should be quick to recognize failure to progress and have a set threshold to convert to an open procedure, should the surgery take longer than is reasonable.

## Patient Positioning

Pediatric patients, particularly infants, are susceptible to crush injuries, and positional injuries. Proper positioning and padding are of paramount importance.

### General Principles

**Figure 1:**
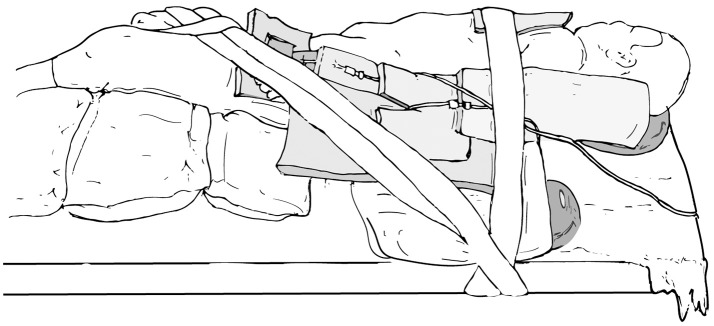
Pad all pressure points and areas around tubing, wires, and nails.

**Figure 2:**
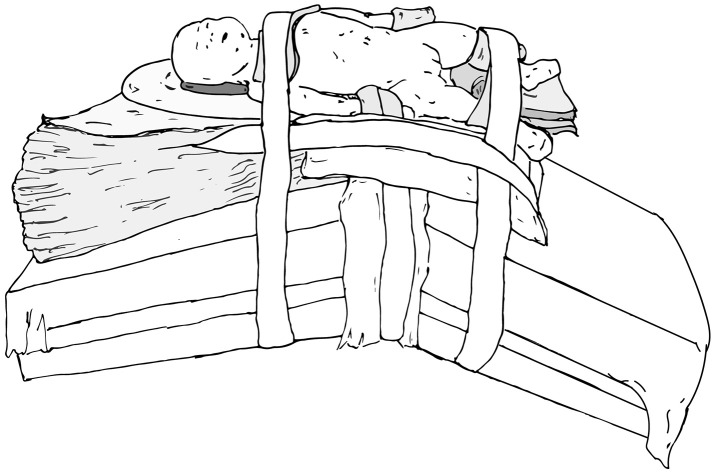
Foam pads are used to elevate small infants (weight < 10 kg) ~10 cm above the operating table to prevent the robot arms from hitting the table.

**Figure 3:**
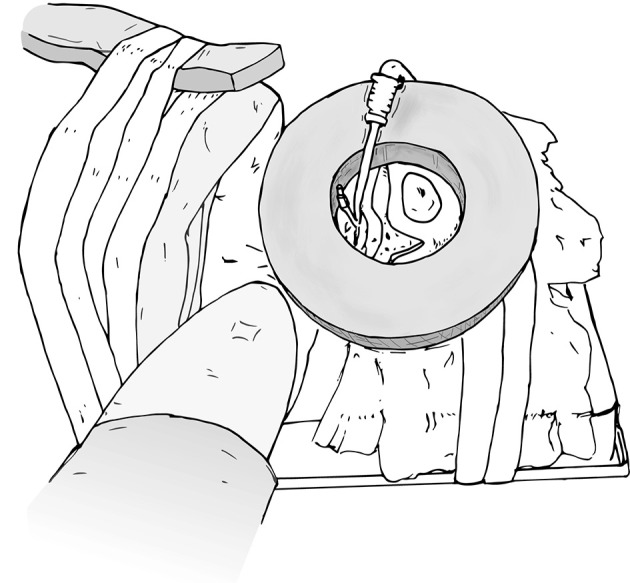
Protect the face with foam to prevent injury or inadvertent manipulation of the endotracheal tube.

The main goals of patient positioning and port placement are to avoid injuries to the patient and to allow maximum mobility of the robotic arms (3). All pressure points and areas around tubing, wires, and nails should be padded (Figure 1). We prefer to use cloth or silk tape and foam. Tape should not be applied directly to exposed skin. Small infants who weigh < 10 kg should be elevated ~10 cm above the operating table using a foam pad to prevent the robot arms from hitting the table (Figure 2). The face should also be protected with foam to prevent injury to the eyes or inadvertent manipulation of the endotracheal tube (Figure 3). Prior to draping the patient, the table should be provoked to the most extreme position expected during the case in order to confirm that the patient is secure, no body part or tubing shifts or kinks, and that there are no anesthetic concerns.

### Upper Urinary Tract Positioning

**Figure 4:**
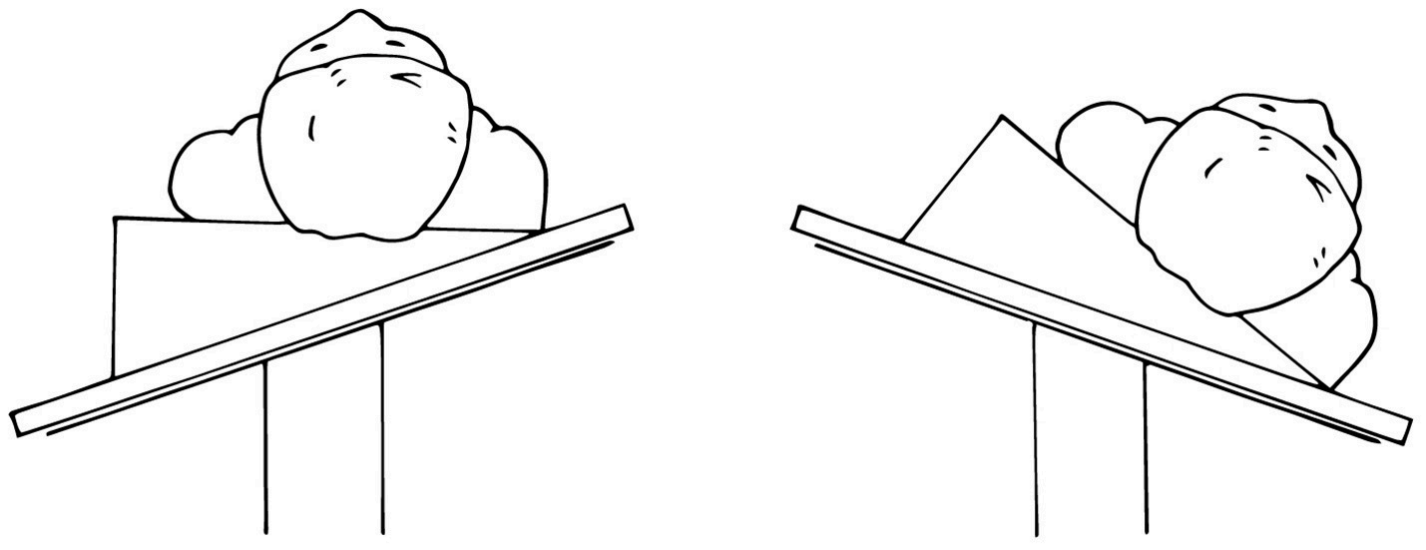
Modified flank positioning used for upper tract surgery, which allows for port placement in a near-supine position (left), and subsequent bowel displacement and renal access via a table tilt (right).

**Figure 5:**
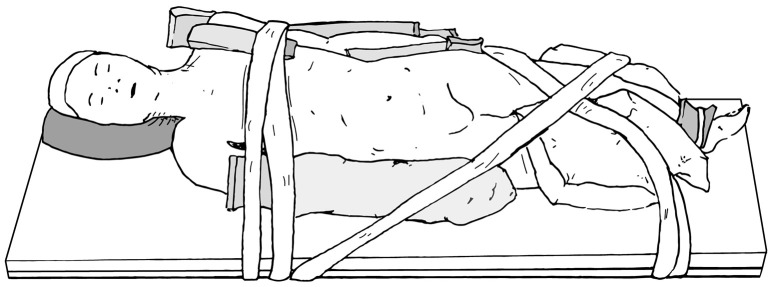
Modified flank positioning used for upper tract surgery, with proper padding, and tape securing the patient to the operating table.

**Figure 6:**
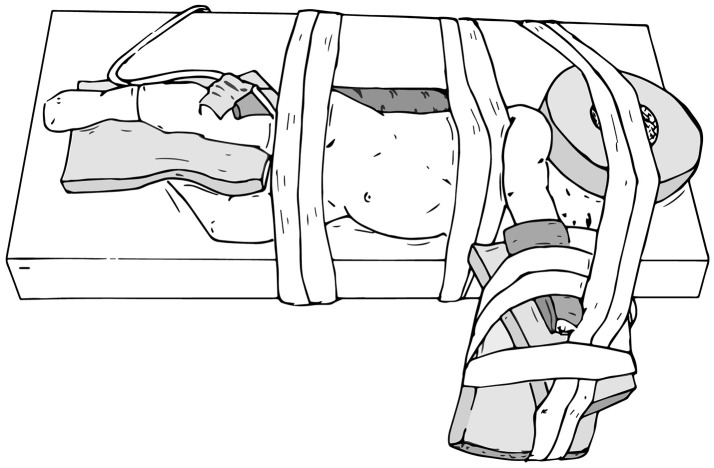
Modified flank positioning with arms placed on an arm board across the body.

When preparing patients for renal and upper tract surgery, we prefer modified flank positioning over lateral decubitus positioning, as the former allows for port placement in a near-supine position, as well as bowel displacement and renal access via a table tilt (Figures 4, 5). For a modified flank position, arms can be placed on an arm board across the body or alongside the patient, depending on surgeon preferences. Figure 6 illustrates the former. While there are many ways to secure the patient, we prefer placing a tape across the forehead in front of the face-foam, across the nipple-line of the chest, as well as diagonally from the arms across the legs on both sides (Figure 5). Egg crates, pillows, and/or an infant-sized bean bag may be used to help support the patient in position.

### Lower Urinary Tract Positioning

**Figure 7:**
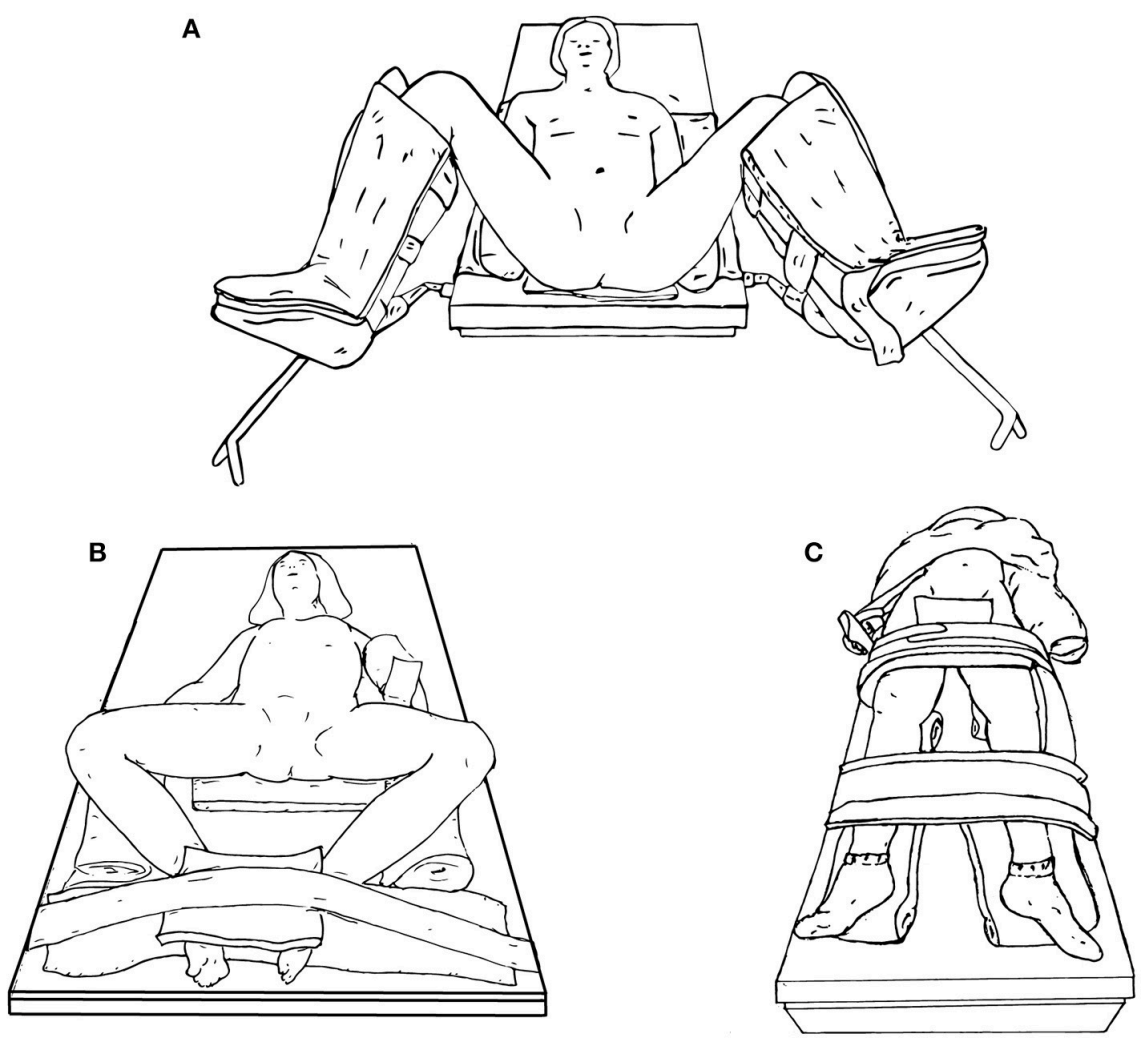
Lower urinary tract surgery positioning options, including lithotomy **(A)**, frog-leg **(B)**, or supine **(C)**.

When positioning a patient for lower urinary tract surgery, the patient may be placed in either lithotomy (Figure 7A), frog-leg position (Figure 7B), or supine (Figure 7C). If cystoscopy is necessary during the procedure, lithotomy is preferred.

## Trocar Placement

Trocar placement is a crucial step in robotic-assisted surgery in order to minimize instrument collision and operative time. A recent study demonstrated that collisions are minimized if the distance between both anterior superior iliac spines is >13 cm, or if the puboxyphoid distance is >15 cm (19).

### Initial Access

Passerotti et al. published the outcomes of 806 laparoscopic procedures (conventional and robot assisted) and reported a 2% complication rate, 75% of which were access related (20). This finding is likely due to the fact that pediatric abdomens are more compliant than adult abdomens, and the pressure necessary to penetrate the pediatric peritoneum can easily cause injury. Therefore, we gain initial access using the open Hassan technique to place the 8.5 mm camera port, always using a blunt black obturator in order to minimize the risk of injury.

### Instrument Ports

Under direct visualization, we employ sharp dissection through the peritoneum with an 11 blade, after injection of 0.25% bupivacaine. Once the port site is dilated with a mosquito clamp, the trocar is advanced with a blunt obturator. While the port is designed to be inserted at the level of the thick black line, in young infants, the port may often only be able to be placed a few mm into the peritoneum.

When using an Si system, there is a longstanding debate regarding the preferred sizes of instrument ports in pediatric patients, as there is a pediatric option with an 8.5 mm camera and 5 mm instruments, compared with the standard option of a 12 mm camera and 8 mm instruments. Although the 5 mm ports require a slightly smaller incision, the 5 mm instruments paradoxically require an additional 2 cm intracorporeal working space due to the design of the instrument arms, as the articulating joints are further back on the 5 mm instruments than they are on the 8 mm instruments (3). In addition, the variety of instruments available in the 5 mm size is greatly limited compared to the 8 mm size, many of which are particularly useful during pediatric reconstruction cases, including Black diamond microforceps, and Potts scissors. We prefer to use a hybrid set with an 8.5 mm pediatric camera as well as the 8 mm instruments. On the Xi system, currently only 8-mm instruments are available.

**Figure 8:**
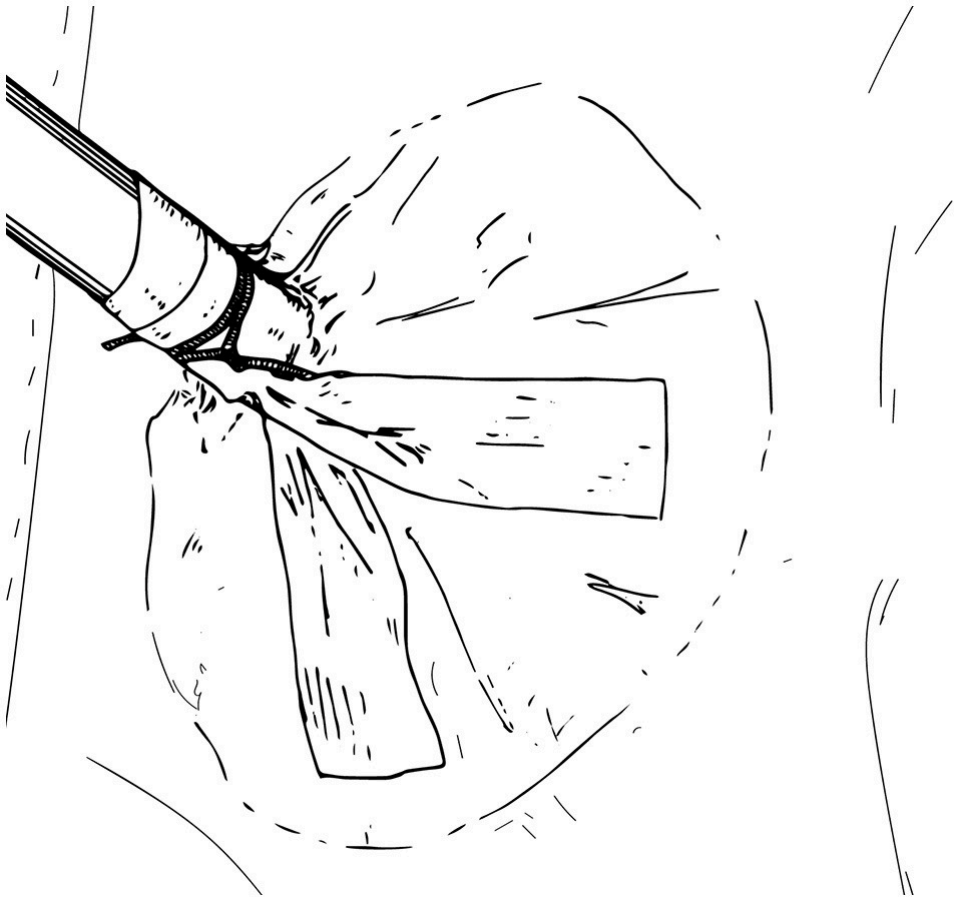
Trocar secured to the skin using sutures, steri-strips, and Tegaderms.

While a 3–5 cm distance between ports is ideal, in infants, this distance is not always possible, so the trocars are just placed as far apart as possible. More distance will be evident after abdominal distension from the pneumoinsufflation. Furthermore, “burping” the ports out 1–2 cm after docking will tent the abdominal wall and also create some extra room. In order to prevent the trocars from falling out, in infants we advocate that they be secured to the skin using a combination of sutures, steri-strips, and Tegaderms (Figure 8). At the conclusion of the case, all ports are closed under direction vision.

### 14-Guage Angiocatheter Assist

A 14-guage angiocatheter in conjunction with cystoscopic tools can be used as an assistant port in order to avoid an additional port placement.

### Trocar Positioning

#### Upper Urinary Tract Trocar Positioning

**Figure 9:**
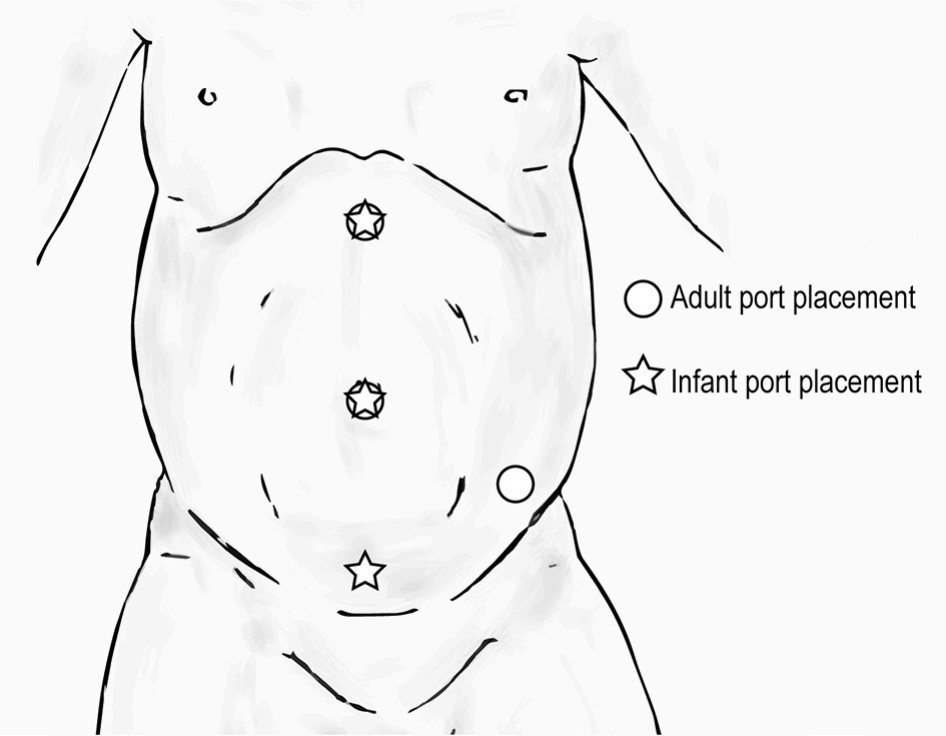
Trocar positioning for upper urinary tract surgery in infants (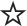) compared to that for adults (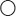).

When performing upper urinary tract surgery such as pyeloplasty or nephrectomy in an infant, in order to prevent robotic arms from clashing, the most inferior port site can be moved medially (Figure 9). This adjustment is also useful when performing a pyeloplasty because a distended ureteropelvic junction can often be found in the lower abdomen in younger children. Of note, with the adjusted location of the most inferior port site, care must be taken to avoid injury to the bladder.

#### Upper Urinary Tract Hidden Incision Endoscopic Surgery (HIdES) Trocar Positioning

**Figure 10:**
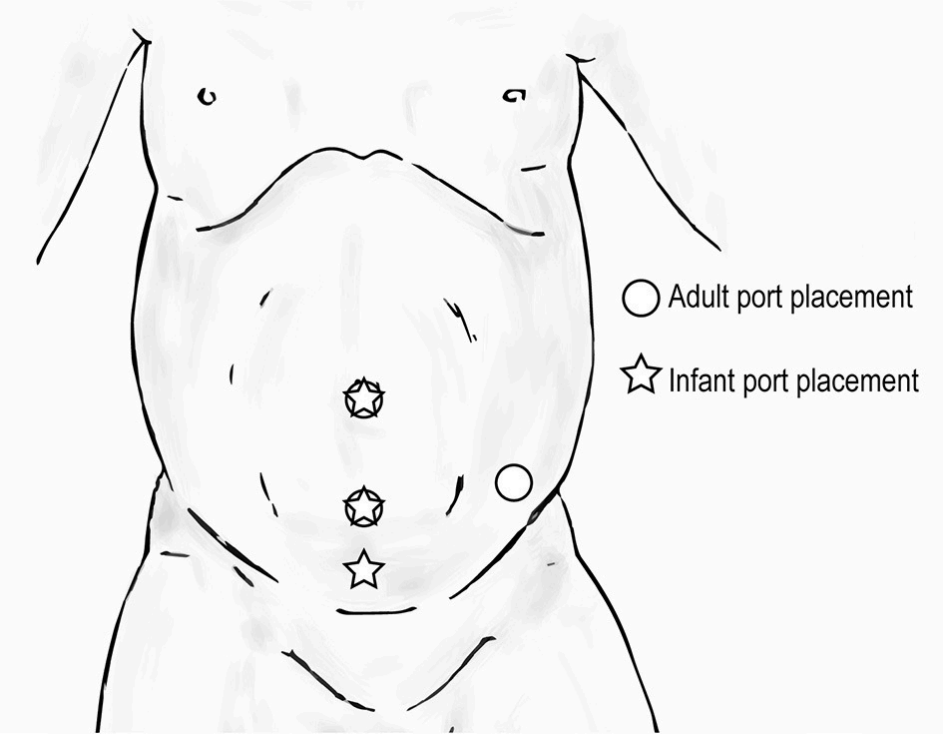
Trocar positioning for upper urinary tract hidden incision endoscopic surgery (HIdES) in infants (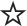) compared to that for adults (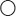).

Hidden incision endoscopic surgery has been previously described for pediatric upper urinary tract surgery (21). The HIdES technique aims to eliminate visible scarring, and the robotic working port and camera port are placed below the line of a Pfannenstiel incision. A second working port is placed infraumbilically. Similar to standard upper urinary tract trocar positioning, the most inferior port can be moved medially in order to increase working room in an infant (Figure 10). Again, care must be taken to avoid injury to the bladder with this adjustment.

#### Extravesical Reimplant

**Figure 11:**
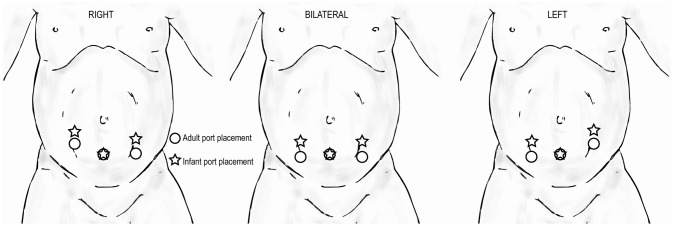
Trocar positioning for extravesical reimplant surgery in infants (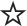) compared to that for adults (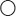) based on laterality of procedure.

When performing extravesical reimplant surgery with robotic assistance, the port site positioning will depend on the laterality of the procedure. If bilateral, the port sites will be in a straight line. For infants, all port sites should be 2 cm above the umbilicus to ensure sufficient working room (Figure 11).

## Docking/Room Set-Up

A standardized room set-up, including positioning of the robot, robotic console, scrub table, anesthesia machines, and surgical assistant, is important to maximize the utility of the robot while maintaining a safe and effective working environment. If using an Si system, we recommend maintaining the robot at one established location in the room and then moving/rotating the operating room table in position in order to minimize robotic manipulation. Communication with anesthesia is paramount, as extension tubing may be necessary. If using an Xi system, the robot arms can be rotated to the desired configuration without manipulating the operating room table.

### Upper Urinary Tract Room Set-Up

**Figure 12:**
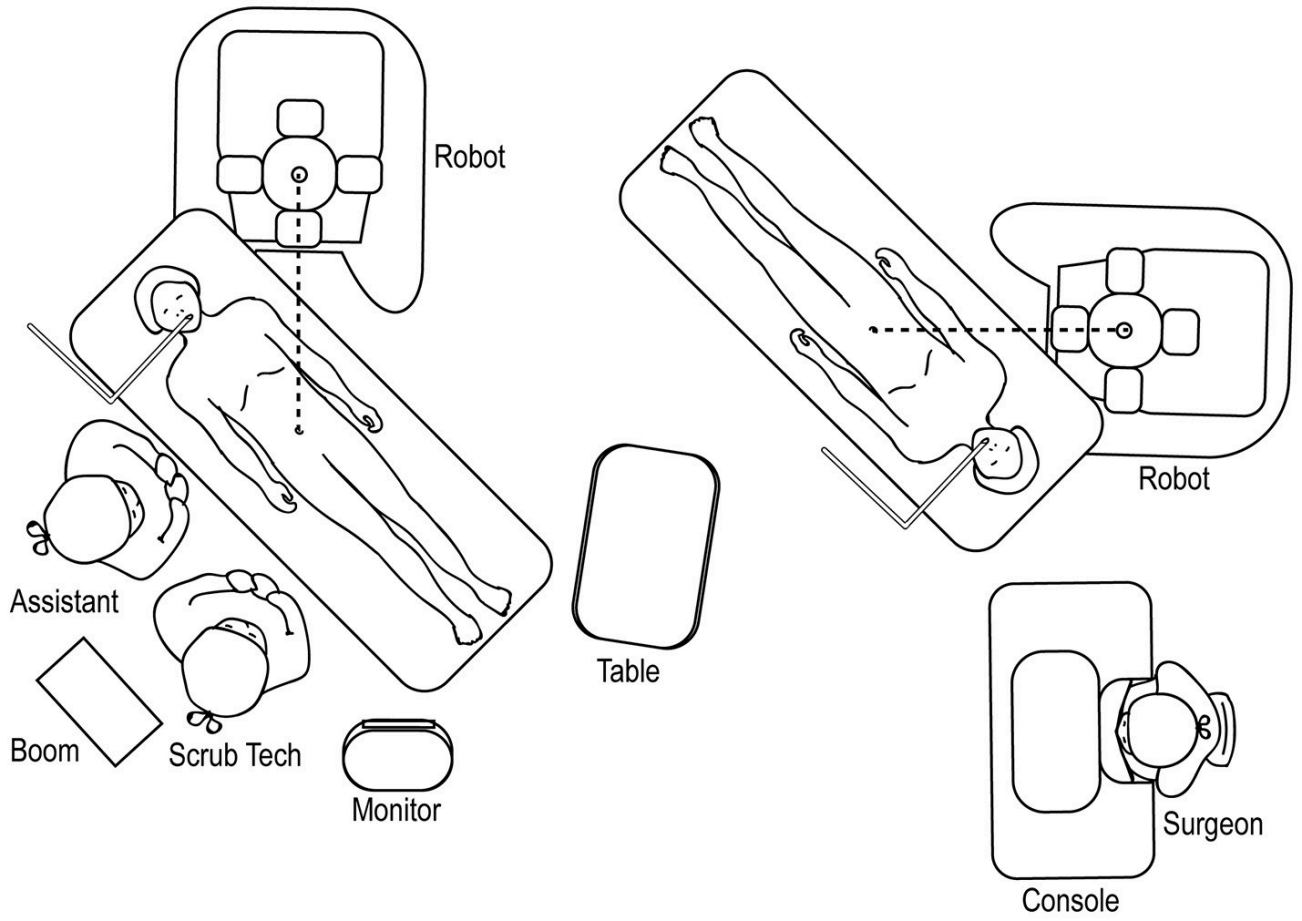
Overhead view of room set-up for upper urinary tract surgery, with robot docked at ipsilateral shoulder.

The robot should be docked at a point that forms a straight line with the patient's umbilicus and the target location of the surgery (Figure 12), identified through either a preoperative retrograde pyelogram (if performing a pyeloplasty) or through direct gross inspection after the initial laparoscopy. The assistant and surgical technician stand on the contralateral side with an accessible table and Mayo stand.

### Upper Urinary Tract Hidden Incision Endoscopic Surgery (HIdES) Room Set-Up

**Figure 13:**
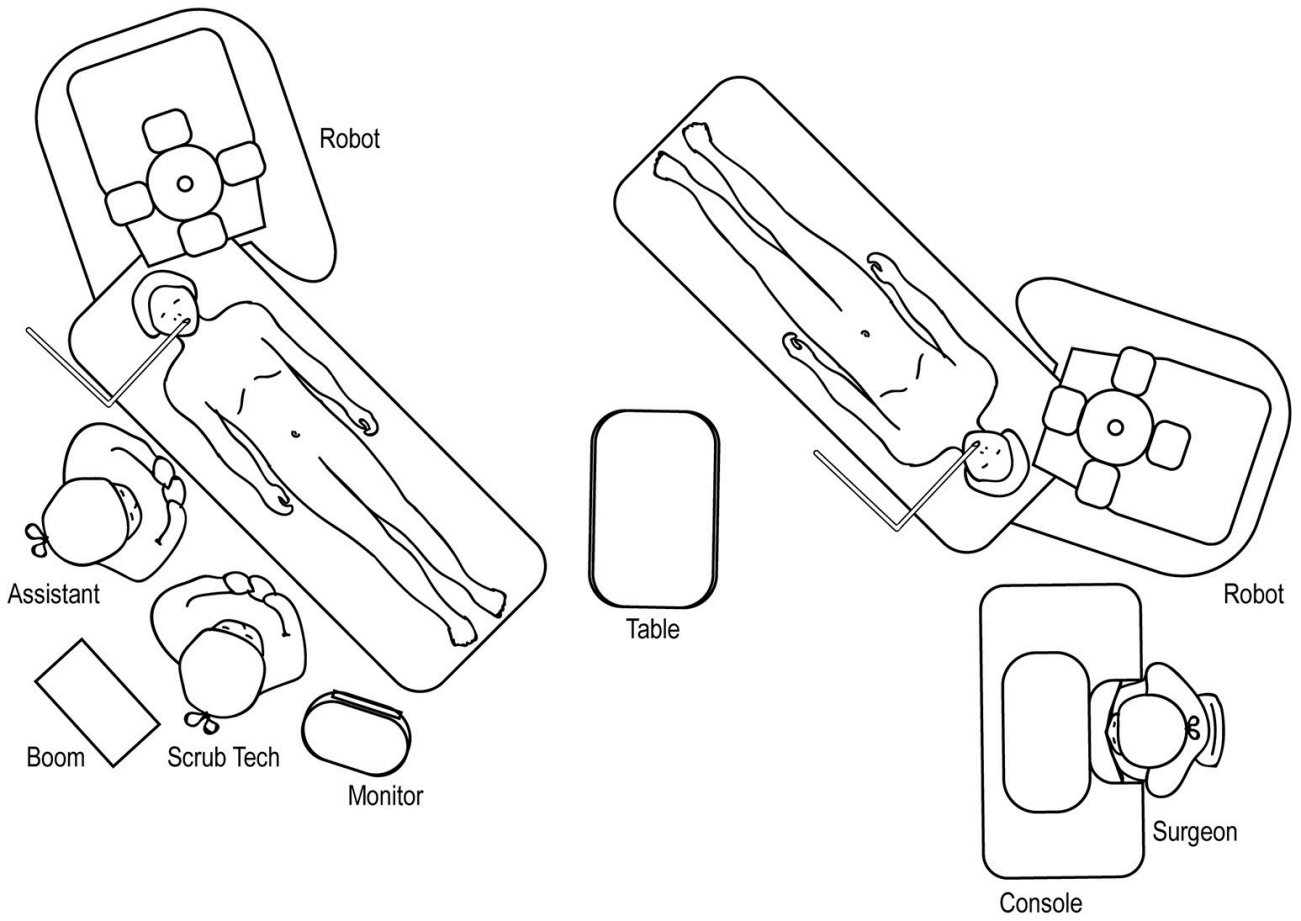
Overhead view of room set-up for upper urinary tract hidden incision endoscopic surgery (HIdES), with robot docked at ipsilateral shoulder.

The room set-up for robotic-assisted upper urinary tract HIdES surgery is similar to that of standard upper urinary tract surgery, except that the robot is docked by coming in from above the ipsilateral shoulder. The feet of the robot should straddle the base of the operating room table (Figure 13).

### Lower Urinary Tract Surgery

**Figure 14:**
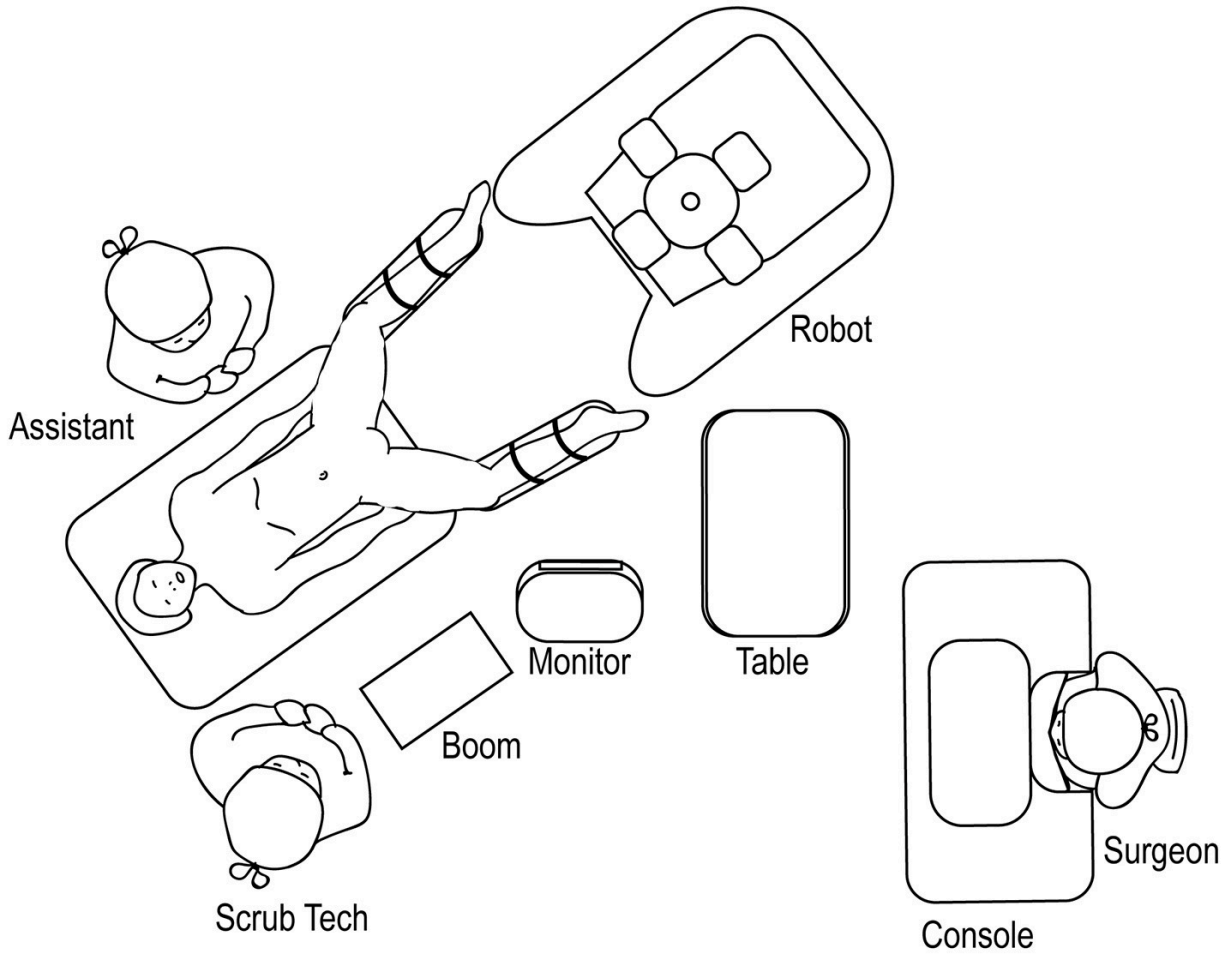
Overhead view of room set-up for lower urinary tract surgery in the lithotomy or frog-leg supine positions.

**Figure 15:**
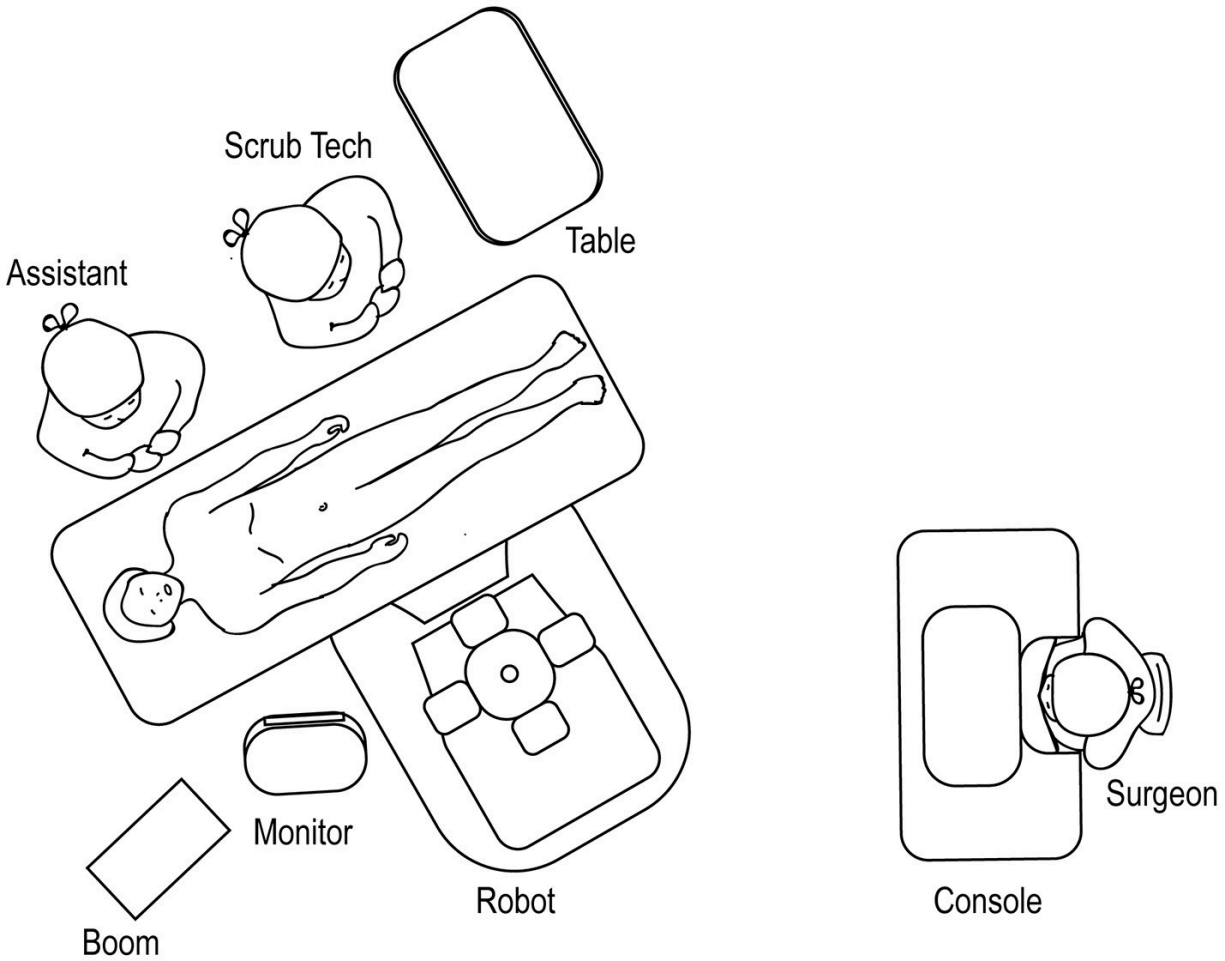
Overhead view of room set-up for lower urinary tract surgery in the supine position.

For robotic-assisted lower urinary tract surgery performed with the patient in the lithotomy or frog-leg supine position, the robot is docked at the patient's 6 o'clock position (Figure 14). The surgical tech, Mayo stand, and scrub table remain on one side of the patient, while the assistant stands at the other. For robotic-assisted lower urinary tract surgery performed with the patient in the supine position, the robot is docked laterally (Figure 15), with the feet of the Si system straddling the base of the table. The assistant and surgical technician remain on the contralateral side of the robot. If using an Xi system, the positioning of the robot is versatile as the arms can be rotated to the appropriate configuration.

## Team Considerations

Robotic surgery is a team effort. A successful program requires dedicated support from the hospital as well as an established nursing team. While infant robotic cases are rarely performed daily, preoperative huddles, and standardized algorithms can be invaluable in helping establish a protocol for staff to follow, minimizing confusion, and variations in practice. Additionally, we recommend contingency plans for potential robotic emergencies. These include bleeding, loss of airway, equipment malfunction, and lost needle. Specific step-by-step plans and allocation of responsibilities should be established and practiced with the team in order to minimize reaction times.

## Conclusion

Young infants warrant special consideration when considering robotic surgery. Their unique anatomy makes them particularly susceptible to the physiologic effects of pneumoperitoneum. When undertaking these cases, surgeons should strive to limit insufflation time and minimize the insufflation pressure to the lowest possible level in order to accomplish the surgery. Furthermore, while the DaVinci robotic system was designed to be used in adult patients, the machine can be used in young children, as long as the specific considerations outlined here are taken into account during positioning, port placement, and docking.

## Author Contributions

SK authored and edited the manuscript. JB edited the manuscript and contributed original illustrations. AA conceived focus of review and edited the manuscript.

### Conflict of Interest Statement

The authors declare that the research was conducted in the absence of any commercial or financial relationships that could be construed as a potential conflict of interest.
